# Different estimation techniques for constant-partially accelerated life tests of chen distribution using complete data

**DOI:** 10.1038/s41598-023-42055-8

**Published:** 2023-09-20

**Authors:** H. M. M. Radwan, Abdulaziz Alenazi

**Affiliations:** 1 Mathematics Department, Faculty of Science, Minia University, Minia, 61519 Egypt; 2 Mathematics Department, College of Science, Northern Border University, Arar, Saudi Arabia

**Keywords:** Mathematics and computing, Statistics

## Abstract

The issue of various estimation techniques in constant partially accelerated life tests with complete data is the main subject of this research. The Chen distribution is regarded as an item’s lifetime under use conditions. To estimate the distribution parameters and the acceleration factor, maximum likelihood estimation, least square estimation, weighted least square estimation, Cramér Von–Mises estimation, Anderson–Darling estimation, right-tail Anderson–Darling estimation, percentile estimation, and maximum product of spacing estimation are presented for classical estimation. For illustrative purposes, two real data sets are analyzed. The investigation of the two real data sets reveals that the suggested techniques are practical and can be used to solve some engineering-related issues. In order to compare the results of the several estimation techniques that have been offered based on mean square error and absolute average bias, a simulation study is presented at the end. When adopting the smallest values for mean square error and absolute average bias, this study demonstrates that maximum product of spacing estimation is the technique that is most effective among the alternatives in most cases.

## Introduction

The rapid and successive technological developments in various engineering fields have led to an increase in the demand for products with high reliability. These products are difficult to test for reliability during the lifetime test period due to the high cost, which makes it difficult to collect failure information under the use conditions. Therefore, accelerated lifetime tests (ALT) are used to collect failure time data for test products in much less time and at a lower cost. In accelerated life tests, the experiment can either start under accelerated conditions and continue under these conditions, or under use conditions and then apply the accelerated conditions to those units that did not fail within the predetermined time under the influence of use conditions. Accordingly, there are two main types of ALT: fully ALT, which are suitable for the first case, and partially accelerated life tests (PALT), which are suitable for the latter case.

One of the most popular models that has received a lot of attention in the literature on fully ALT is the constant accelerated life tests (CALT). Numerous statisticians have addressed the issue of various estimation techniques based on CALT using complete data, including^1–5^.

The two most significant PALT models in the literature are step-stress PALT and constant (CPALT). Goel^6^ investigated, in a step-stress PALT, the MLE technique for parameter estimation for exponential and uniform distributions utilizing complete data. To estimate the exponential distribution’s parameters in a step-stress PALT with complete data, Bayesian technique was utilized, see^7^.

According to^8^, the CPALT only performs each item under the use or accelerated conditions. Additionally, the authors used type-I censored data to investigate the CPALT issue for exponential distributions. Subsequent studies addressed the issue of CPALT estimation under various censored data utilizing various distributions. Using CPALT with type-I censored data, Bai et al.^9^ investigated the of estimation and optimal design for the log-normal distribution. Abdel-Hamid^10^ investigated the problem of estimation for Burr type-II distribution based on CPALT using progressive type-II. In CPALT, the optimal design problem for the inverse Weibull distribution utilizing type-I censored data has been studied by^11^. Using a unified hybrid censoring data, Lone et al.^12^ have taken into account the prediction issue in CPALT for the Gompertz distribution. The estimation for the two-parameter Gompertz distribution using CPALT under an adaptive progressive hybrid censoring scheme was introduced in^13^. In accordance with an adaptive type-II progressive censoring scheme, Almalki et al.^14^ introduced parameter estimation for the Kumaraswamy distribution using CPALT. Mahmoud et al.^15^ proposed parameter estimation for the inverted generalized linear exponential distribution under CPALT using a progressive type-II censoring scheme. parameter estimation for the Nadarajah–Haghighi distribution based on the progressive type-II censoring scheme was investigated by^16^. For the modified Kies exponential distribution, Nassar and Alam^17^ examined parameter estimation based on the CPALT utilizing a type-II censoring data. Based on progressive first failure type-II censored using CPALT, Eliwa and Ahmed^18^ conducted a reliability analysis of the Lomax model.

On the other hand, the issue of various estimation techniques based on CPALT using complete data, which is the focus of this study, has not been adequately addressed. Additionally, a variety of natural phenomena, engineering problems, and clinical treatment produce a large amount of complete real data that are extremely important to our life. According to the aforementioned, the issue of various estimation methodologies based on CPALT and employing complete real data is of considerable relevance.

With an increasing or bathtub-shaped hazard rate function (HRF), Chen^19^ suggested a two-parameter lifetime distribution. Due to the fact that the bathtub-shaped HRF serves as a useful conceptual model for electronic and machinery industries, it has received consideration from numerous researchers; see^20–23^.

The Chen distribution has some distinctive properties compared to other models with two parameters such as the fact that its HRF is bathtub-shaped and also the confidence intervals for the shape parameter and the joint confidence regions for the two parameters have closed form. Therefore, many researchers have studied its statistical inference based on ALT and PALT, see^24–26^.

The main objective of this research is to provide eight techniques of estimations for CPALT of Chen distribution based on the complete data, namely: maximum likelihood estimation (MLE), least square estimation (LSE), weighted least square estimation (WLSE), Cramér Von–Mises estimation (CVME), Anderson–Darling estimation (ADE), right-tail Anderson–Darling estimation (RADE), percentile estimation (PE), and maximum product of spacing estimation (MPSE). To illustrate the importance of the model in resolving various engineering issues, two complete real data sets are used. A simulation study is conducted to assess the performance of the suggested methods. Small, medium, and large sample sizes were used to compare the mean squared errors (MSE) and the absolute average bias (AAB) of the estimators’ performances.

The sections of this study are arranged as follows. “Basic assumptions and model description” presents the main concepts of CPALT. The MLE of Chen distribution using CPALT is studied in “Maximum likelihood estimation”. “Least square and weighted least square estimations” discusses the estimation of Chen distribution using CPALT based on LSE and WLSE. The CVME is studied for Chen distribution under CPALT in “Cramér Von–Mises estimation”. In “Anderson–Darling and right-tail Anderson–Darling estimations”, the ADE and RTADE are presented to estimate the unknown parameters and accelerated factor of Chen distribution using CPALT. PE using CPALT for Chen distribution is studied in “Percentile estimation”. In “Maximum product of spacing estimation”, the MPSE is presented for Chen distribution under CPALT. Two uncensored real data sets are analyzed in “Numerical computations”. In “Simulation study”, the simulation study is covered. Conclusion remarks are reported in “Conclusion”.

## Basic assumptions and model description

### Basic assumptions

- Under use conditions, the lifetimes of test units follow the Chen distribution and are independent and identically distributed. The probability density function (PDF) and the cumulative distribution function (CDF) of Chen distribution are given by
  1 $$\begin{aligned} f_{1}(t;\lambda ,\xi ) =\lambda \ \xi \ e^{t^{\xi }} \ t^{\xi -1} \ e^{\lambda (1-e^{t^{\xi }})}, \qquad \lambda>0, \ \xi>0, \ t>0. \end{aligned}$$
  and
  2 $$\begin{aligned} F_{1}(t;\lambda ,\xi ) = 1- e^{\lambda (1-e^{t^{\xi }})}, \qquad t>0. \end{aligned}$$
  respectively. The survival function (SF) and HRF are given by:
  3 $$\begin{aligned} S_{1}(t;\lambda ,\xi ) =e^{\lambda (1-e^{t^{\xi }})}, \end{aligned}$$
  and
  4 $$\begin{aligned} h_{1}(t;\lambda ,\xi ) = \lambda \ \xi \ e^{t^{\xi }} \ t^{\xi -1}. \end{aligned}$$
  respectively. Equation (4) can be used to demonstrate how the hazard rate function for the Chen distribution can have two different shapes: an increasing shape for $\xi \ge 1$ and $\lambda >0$ and a bathtub shape for $\xi <1$ and $\lambda >0$ with change point at $t^*=\left( \frac{1-\xi }{\xi }\right) ^{1/\xi }$. For additional properties, see^27^.
- Under the acceleration condition, the lifetimes of test units follow the Chen distribution and are independent and identically distributed. The HRF of test unit can be given by $h_{2}(t)=\varphi \ h_{1}(t)$, where $\varphi >1$ is the acceleration factor. Then the HRF, SF, CDF, and PDF can be written as
  5 $$\begin{aligned} h_{2}(t;\lambda ,\xi ,\varphi )= & {} \lambda \ \varphi \ \xi \ e^{t^{\xi }} \ t^{\xi -1}, \end{aligned}$$
  6 $$\begin{aligned} S_{2}(t;\lambda ,\xi ,\varphi )= & {} e^{\int _{0}^{t}h_{2}(x;\lambda ,\xi ,\varphi )}=e^{\lambda \ \varphi (1-e^{t^{\xi }})}, \end{aligned}$$
  7 $$\begin{aligned} F_{2}(t;\lambda ,\xi ,\varphi )= & {} 1- e^{\lambda \ \varphi (1-e^{t^{\xi }})}, \end{aligned}$$
  and
  8 $$\begin{aligned} f_{2}(t;\lambda ,\xi ,\varphi ) = \lambda \ \xi \ \varphi \ e^{t^{\xi }} \ t^{\xi -1} \ e^{\lambda \ \varphi (1-e^{t^{\xi }})}, \end{aligned}$$

respectively.

### Model description

Upon using the CPALT, the total size of units is divided into two groups: $m_{1}$ units for use condition and $m_{2}$ units for accelerated condition. Let the lifetime $T_{ji}$, $i=1,\ldots ,m_{j}$, $j=1,2$ be two complete samples from Chen distribution. The lifetime of an item tested at use conditions is denoted by $T_{1i}$, while the lifetime of an item tested at accelerated conditions is denoted by $T_{2i}$. The two lifetimes $T_{1i}$ and $T_{2i}$ are pairwise statistically independent.

## Point estimation

The MLE, LSE, WLSE, CVME, ADE, RADE, PE, and MPSE estimation methods under CPALT for Chen distribution utilizing complete data are explored in this section.

### Maximum likelihood estimation

The MLEs of parameters and accelerated factor of Chen distribution under complete data using CPALT are discussed in this subsection. The likelihood function of CPALT for Chen distribution under complete data can be obtained as:

9 $$\begin{aligned} L(\lambda ,\xi ,\varphi |{\textbf {t}})= \prod _{j=1}^{2} \ \prod _{i=1}^{m_{j}} \ f_{j}(t_{ji};\lambda , \xi , \varphi ). \end{aligned}$$

Upon using (1) and (8), the log-likelihood function $\ell =\log \ L(\lambda ,\xi ,\varphi |{\textbf {t}})$ can be written as

10 $$\begin{aligned} \ell (\lambda ,\xi ,\varphi |{\textbf {t}})= & {} (m_{1}+m_{2}) \log {\lambda }+(m_{1}+m_{2}) \log { \xi }+m_{2}\log {\varphi } +(\xi -1)\sum _{i=1}^{m_{1}} \ \log {t_{1i}}+\sum _{i=1}^{m_{1}} \ t_{1i}^\xi \nonumber \\{} & {} \,+ \sum _{i=1}^{m_{1}} \ \lambda \ (1-e^{t_{1i}^\xi }) + (\xi -1)\sum _{i=1}^{m_{2}} \ \log {t_{2i}}+\sum _{i=1}^{m_{2}} \ t_{2i}^\xi +\sum _{i=2}^{m_{2}} \ \lambda \ \varphi \ (1-e^{t_{2i}^\xi }). \end{aligned}$$

The normal equations of the unknown parameters and the accelerated factor can be given as:

11 $$\begin{aligned} \frac{\partial \ell }{\partial \lambda }= & {} \frac{m_{1}+m_{2}}{\lambda }+\sum _{i=1}^{m_{1}} \ (1-e^{t_{1i}^\xi })+ \sum _{i=1}^{m_{2}} \ \varphi \ (1-e^{t_{2i}^\xi })=0, \end{aligned}$$

12 $$\begin{aligned} \frac{\partial \ell }{\partial \xi }= & {} \frac{m_{1}+m_{2}}{\xi }+\sum _{i=1}^{m_{1}} \ \log {t_{1i}}+\sum _{i=1}^{m_{1}} \ t_{1i}^\xi \ \log {t_{1i}} -\sum _{i=1}^{m_{1}} \ \lambda \ t_{1i}^\xi \ \log {t_{1i}} \ e^{t_{1i}^\xi } \nonumber \\{} & {} \,+ \sum _{i=1}^{m_{2}} \ \log {t_{2i}}+\sum _{i=1}^{m_{2}} \ t_{2i}^\xi \ \log {t_{2i}} -\sum _{i=1}^{m_{2}} \ \lambda \ \varphi \ t_{2i}^\xi \ \log {t_{2i}} \ e^{t_{2i}^\xi } =0, \end{aligned}$$

and

13 $$\begin{aligned} \frac{\partial \ell }{\partial \varphi }= & {} \frac{m_{2}}{\varphi }+\sum _{i=1}^{m_{2}} \ \lambda \ (1-e^{t_{2i}^\xi })=0. \end{aligned}$$

The three aforementioned equations do not have a closed-form solution, hence the MLEs can be obtained using a numerical methodology by employing **FindRoot[]** in ** Mathematica** or by using Newton–Raphson algorithm. The MLEs of $\lambda$, $\xi$, and $\varphi$ can be denoted as ${\hat{\lambda }}$, ${\hat{\xi }}$, and ${\hat{\varphi }}$.

### Least square and weighted least square estimations

LSE and WLSE are presented for estimating the beta distribution’s parameters in the paper by^28^. With the use of CPALT, these techniques will be used to estimate the unknown parameters and the accelerated factor of Chen distribution under complete data. For this purpose, take lifetimes $T_{(ji)}$, $i=1,\ldots ,m_{j}$, $j=1,2$ to be two complete ordered samples from Chen distribution under CPALT. Then the LSE of the unknown parameters and the accelerated factor can be obtained by minimizing the following function

14 $$\begin{aligned} S= \sum _{j=1}^2 \ \sum _{i=1}^{m_{j}} \left( \ F_{j}(t_{(ji)};\lambda , \xi , \varphi )-\frac{ i}{ m_{j}+1} \right) ^2 \ \end{aligned}$$

w.r.t. the unknown parameters $\lambda$, $\xi$, and the accelerated factor $\varphi$. Upon using (2) and (7), the equation (14) can be written as

$$\begin{aligned} S= \sum _{i=1}^{m_{1}} \left( 1- e^{\lambda \left( \ 1-e^{t_{(1i)}^{\xi }}\right) \ }-\frac{ i}{ m_{1}+1} \right) ^2 \ +\sum _{i=1}^{m_{2}} \left( 1- e^{\lambda \ \varphi \left( \ 1-e^{t_{(2i)}^{\xi }}\right) \ }-\frac{ i}{ m_{2}+1} \right) ^2 \ \end{aligned}$$

Additionally, the following non-linear equations can be solved to yield the LSEs of the unknown parameters and the accelerated factor.

$$\begin{aligned} \frac{\partial S}{\partial \lambda }= & {} \sum _{i=1}^{m_{1}} \ \left( 1- e^{\lambda \left( 1-e^{t_{(1i)}^{\xi }}\right) \ }-\frac{ i}{ m_{1}+1} \right) \ \delta _{1}(t_{(1i)};\lambda , \xi ) \\{} & {} \,+\sum _{i=1}^{m_{2}} \ \left( 1- e^{\lambda \ \varphi \left( \ 1-e^{t_{(2i)}^{\xi }}\right) \ }-\frac{ i}{ m_{2}+1} \right) \ \delta _{2}(t_{(2i)};\lambda , \xi , \varphi ) =0, \\ \frac{\partial S}{\partial \xi }= & {} \sum _{i=1}^{m_{1}} \ \left( 1- e^{\lambda \left( 1-e^{t_{(1i)}^{\xi }}\right) \ }-\frac{ i}{ m_{1}+1} \right) \ \delta _{3}(t_{(1i)};\lambda , \xi )\\{} & {} \,+\sum _{i=1}^{m_{2}} \ \left( 1- e^{\lambda \ \varphi \left( \ 1-e^{t_{(2i)}^{\xi }}\right) \ }-\frac{ i}{ m_{2}+1} \right) \ \delta _{4}(t_{(2i)};\lambda , \xi , \varphi ) =0, \end{aligned}$$

and

$$\begin{aligned} \frac{\partial S}{\partial \varphi }= \sum _{i=1}^{m_{2}} \ \left( \ 1- e^{\lambda \ \varphi \left( 1-e^{t_{(2i)}^{\xi }}\right) \ }-\frac{ i}{ m_{2}+1} \right) \ \delta _{5}(t_{(2i)};\lambda , \xi , \varphi )=0, \end{aligned}$$

where

15 $$\begin{aligned} \delta _{1}(t_{(1i)};\lambda , \xi )=- e^{\lambda \left( \ 1-e^{t_{(1i)}^{\xi }}\right) \ } \left( 1-e^{t_{(1i)}^{\xi }}\right) \, \end{aligned}$$

16 $$\begin{aligned} \delta _{2}(t_{(2i)};\lambda , \xi , \varphi )=-\varphi \ e^{\lambda \ \varphi \left( 1-e^{t_{(2i)}^{\xi }}\right) \ } \left( \ 1-e^{t_{(2i)}^{\xi }}\right) \, \end{aligned}$$

17 $$\begin{aligned} \delta _{3}(t_{(1i)};\lambda , \xi )= e^{\lambda \left( \ 1-e^{t_{(1i)}^{\xi }}\right) \ } \ \lambda \ t_{(1i)}^\xi \ \log {t_{(1i)}} \ e^{t_{(1i)}^\xi }, \end{aligned}$$

18 $$\begin{aligned} \delta _{4}(t_{(2i)};\lambda , \xi , \varphi )= e^{\lambda \ \varphi \left( 1-e^{t_{(2i)}^{\xi }}\right) \ } \ \lambda \ \varphi \ t_{(1i)}^\xi \ \log {t_{(1i)}} \ e^{t_{(1i)}^\xi }, \end{aligned}$$

and

19 $$\begin{aligned} \delta _{5}(t_{(2i)};\lambda , \xi , \varphi )= -\lambda \ e^{\lambda \ \varphi \left( 1-e^{t_{(2i)}^{\xi }}\right) \ } \left( \ 1-e^{t_{(2i)}^{\xi }}\right) . \end{aligned}$$

One can obtain WLSEs for Chen distribution using CPALT under complete data by minimizing the following function

$$\begin{aligned} W=\sum _{j=1}^2 \ \sum _{i=1}^{m_{j}} \ \frac{ (m_{j} + 2) (m_{j} + 1)^2}{i \ (m_{j} - i + 1)} \ \left( \ F_{j}(t_{(ji)};\lambda , \xi , \varphi )-\frac{ i}{ m_{j}+1} \right) ^2 \end{aligned}$$

w.r.t. the unknown parameters $\lambda$, $\xi$, and the accelerated factor $\varphi$ or by finding the solution to the non-linear equations

$$\begin{aligned} \frac{\partial W}{\partial \lambda }= & {} \sum _{i=1}^{m_{1}} \ \frac{ (m_{1} + 2) (m_{1} + 1)^2}{i \ (m_{1} - i + 1)} \ \left( 1- e^{\lambda \left( 1-e^{t_{(1i)}^{\xi }}\right) \ }-\frac{ i}{ m_{1}+1} \right) \ \delta _{1}(t_{(1i)};\lambda , \xi ) \\{} & {} \, + \sum _{i=1}^{m_{2}} \ \frac{ (m_{2} + 2) (m_{2} + 1)^2}{i \ (m_{2} - i + 1)} \ \left( 1- e^{\lambda \ \varphi \left( \ 1-e^{t_{(2i)}^{\xi }}\right) \ }-\frac{ i}{ m_{2}+1} \right) \ \delta _{2}(t_{(2i)};\lambda , \xi , \varphi ) =0, \\ \frac{\partial W}{\partial \xi }= & {} \sum _{i=1}^{m_{1}} \ \frac{ (m_{1} + 2) (m_{1} + 1)^2}{i \ (m_{1} - i + 1)} \ \left( 1- e^{\lambda \left( 1-e^{t_{(1i)}^{\xi }}\right) \ }-\frac{ i}{ m_{1}+1} \right) \ \delta _{3}(t_{(1i)};\lambda , \xi )\\ {}{} & {} \, +\sum _{i=1}^{m_{2}} \ \frac{ (m_{2} + 2) (m_{2} + 1)^2}{i \ (m_{2} - i + 1)} \ \left( \ 1- e^{\lambda \ \varphi \left( 1-e^{t_{(2i)}^{\xi }}\right) \ }-\frac{ i}{ m_{2}+1} \right) \ \delta _{4}(t_{(2i)};\lambda , \xi , \varphi ) =0, \end{aligned}$$

and

$$\begin{aligned} \frac{\partial W}{\partial \varphi }= \sum _{i=1}^{m_{2}} \ \frac{ (m_{2} + 2) (m_{2} + 1)^2}{i \ (m_{2} - i + 1)} \ \left( \ 1- e^{\lambda \ \varphi \left( 1-e^{t_{(2i)}^{\xi }}\right) \ }-\frac{ i}{ m_{2}+1} \right) \ \delta _{5}(t_{(2i)};\lambda , \xi , \varphi )=0, \end{aligned}$$

where $\delta _{1}(t_{(1i)};\lambda , \xi )$, $\delta _{2}(t_{(2i)};\lambda , \xi , \varphi )$, $\delta _{3}(t_{(1i)};\lambda , \xi )$, $\delta _{4}(t_{(2i)};\lambda , \xi , \varphi )$ and $\delta _{5}(t_{(2i)};\lambda , \xi , \varphi )$ are given by (15), (16), (17), (18) and (19) respectively.

### Cramér Von–Mises estimation

A study by^29^ revealed that the bias of CVME is lower than that of the other minimum distance estimator. In this subsection, CVME is applied to Chen distribution using CPALT based on complete data. Then the CVME of the unknown parameters and the accelerated factor can be given by minimizing the following function

$$\begin{aligned} C=\frac{1}{12 \ (m_{1}+m_{2})} +\sum _{j=1}^2 \ \sum _{i=1}^{m_{j}} \left( F_{j}(t_{(ji)};\lambda , \xi , \varphi )-\frac{2 \ i-1}{2 \ m_{j}} \right) ^2 \ \end{aligned}$$

w.r.t. the unknown parameters $\lambda$, $\xi$, and the accelerated factor $\varphi$ or by solving the following non-linear equations

$$\begin{aligned} \frac{\partial C}{\partial \lambda }= & {} \sum _{i=1}^{m_{1}} \ \left( 1- e^{\lambda \left( \ 1-e^{t_{(1i)}^{\xi }}\right) \ }-\frac{2 \ i-1}{2 \ m_{1}} \right) \ \delta _{1}(t_{(1i)};\lambda , \xi )\\{} & {} \,+\sum _{i=1}^{m_{2}} \ \left( 1- e^{\lambda \ \varphi \left( 1-e^{t_{(2i)}^{\xi }}\right) \ }-\frac{2 \ i-1}{2 \ m_{2}} \right) \ \delta _{2}(t_{(2i)};\lambda , \xi , \varphi ) =0, \\ \frac{\partial C}{\partial \xi }= & {} \sum _{i=1}^{m_{1}} \ \left( 1- e^{\lambda \left( 1-e^{t_{(1i)}^{\xi }}\right) \ }-\frac{2 \ i-1}{2 \ m_{1}} \right) \ \delta _{3}(t_{(1i)};\lambda , \xi )\\{} & {} \, +\sum _{i=1}^{m_{2}} \ \left( 1- e^{\lambda \ \varphi \left( 1-e^{t_{(2i)}^{\xi }}\right) \ }-\frac{2 \ i-1}{2 \ m_{2}} \right) \ \delta _{4}(t_{(2i)};\lambda , \xi , \varphi ) =0, \end{aligned}$$

and

$$\begin{aligned} \frac{\partial C}{\partial \varphi }= \sum _{i=1}^{m_{2}} \ \left( 1- e^{\lambda \ \varphi \left( 1-e^{t_{(2i)}^{\xi }}\right) \ }-\frac{2 \ i-1}{2 \ m_{2}} \right) \ \delta _{5}(t_{(2i)};\lambda , \xi , \varphi )=0, \end{aligned}$$

where $\delta _{1}(t_{(1i)};\lambda , \xi )$, $\delta _{2}(t_{(2i)};\lambda , \xi , \varphi )$, $\delta _{3}(t_{(1i)};\lambda , \xi )$, $\delta _{4}(t_{(2i)};\lambda , \xi , \varphi )$ and $\delta _{5}(t_{(2i)};\lambda , \xi , \varphi )$ are given by (15), (16), (17), (18) and (19) respectively.

### Anderson–Darling and right-tail Anderson–Darling estimations

As an alternative to previous statistical tests for identifying sample distributions deviating from normality, Anderson and Darling^30^ created the Anderson–Darling test. Boos^31^ examined the characteristics of ADE in a different investigation. Using his results, the ADE of Chen distribution using CPALT can be obtained by minimizing the following function

$$\begin{aligned} A= -(m_{1}+m_{2})-\sum _{j=1}^2 \ \sum _{i=1}^{m_{j}} \ \frac{(2 \ i-1)}{m_{j}} \left( \log {F_{j}(t_{(ji)};\lambda , \xi , \varphi )} - \log { \left( 1-F_{j}(t_{(m_{j}+1-ji)};\lambda , \xi , \varphi ) \right) \ } \right) \, \end{aligned}$$

w.r.t. the unknown parameters $\lambda$, $\xi$, and the accelerated factor $\varphi$ or by solving the following non-linear equations:

$$\begin{aligned} \frac{\partial A}{\partial \lambda }= & {} - \sum _{i=1}^{m_{1}} \frac{(2 \ i-1)}{m_{1}} \ \left( \ \frac{\delta _{1}(t_{(1i)};\lambda , \xi )}{ 1- e^{\lambda \left( \ 1-e^{t_{(1i)}^{\xi }} \right) \ }} -\frac{\delta _{1}(t_{(m_{1}+1-1i)};\lambda , \xi )}{ e^{\lambda \left( 1-e^{t_{(m_{1}+1-1i)}^{\xi }} \right) \ }} \right) \ \\ {}{} & {} \, - \sum _{i=1}^{m_{2}} \frac{(2 \ i-1)}{m_{2}} \ \left( \ \frac{\delta _{2}(t_{(2i)};\lambda , \xi , \varphi )}{ 1- e^{\lambda \ \varphi \left( 1-e^{t_{(2i)}^{\xi }} \right) \ }} -\frac{\delta _{2}(t_{(m_{2}+1-2i)};\lambda , \xi , \varphi )}{ e^{\lambda \ \varphi \left( 1-e^{t_{(m_{2}+1-2i)}^{\xi }} \right) \ }} \right) \ =0, \\ \frac{\partial A}{\partial \xi }= & {} - \sum _{i=1}^{m_{1}} \frac{(2 \ i-1)}{m_{1}} \ \left( \ \frac{\delta _{3}(t_{(1i)};\lambda , \xi )}{ 1- e^{\lambda \left( \ 1-e^{t_{(1i)}^{\xi }} \right) \ }} -\frac{\delta _{3}(t_{(m_{1}+1-1i)};\lambda , \xi )}{ e^{\lambda \left( 1-e^{t_{(m_{1}+1-1i)}^{\xi }} \right) \ }} \right) \ \\ {}{} & {} \, - \sum _{i=1}^{m_{2}} \frac{(2 \ i-1)}{m_{2}} \ \left( \ \frac{\delta _{4}(t_{(2i)};\lambda , \xi , \varphi )}{ 1- e^{\lambda \ \varphi \left( 1-e^{t_{(2i)}^{\xi }} \right) \ }} -\frac{\delta _{4}(t_{(m_{2}+1-2i)};\lambda , \xi , \varphi )}{ e^{\lambda \ \varphi \left( 1-e^{t_{(m_{2}+1-2i)}^{\xi }} \right) \ }} \right) \ =0, \end{aligned}$$

and

$$\begin{aligned} \frac{\partial A}{\partial \varphi }=- \sum _{i=1}^{m_{2}} \frac{(2 \ i-1)}{m_{2}} \ \left( \ \frac{\delta _{5}(t_{(2i)};\lambda , \xi , \varphi )}{ 1- e^{\lambda \ \varphi \left( 1-e^{t_{(2i)}^{\xi }} \right) \ }} -\frac{\delta _{5}(t_{(m_{2}+1-2i)};\lambda , \xi , \varphi )}{ e^{\lambda \ \varphi \left( 1-e^{t_{(m_{2}+1-2i)}^{\xi }} \right) \ }} \right) \ =0, \end{aligned}$$

where $\delta _{1}(t_{(1i)};\lambda , \xi )$, $\delta _{2}(t_{(2i)};\lambda , \xi , \varphi )$, $\delta _{3}(t_{(1i)};\lambda , \xi )$, $\delta _{4}(t_{(2i)};\lambda , \xi , \varphi )$ and $\delta _{5}(t_{(2i)};\lambda , \xi , \varphi )$ are given by (15), (16), (17), (18) and (19), respectively.

The RTADE of Chen distribution using CPALT can be given by minimizing the following function

$$\begin{aligned} R= \frac{(m_{1}+m_{2})}{2}-2 \ \sum _{j=1}^2 \ \sum _{i=1}^{m_{j}} \ F_{j}(t_{(ji)};\lambda , \xi , \varphi ) - \sum _{j=1}^2 \ \sum _{i=1}^{m_{j}} \ \frac{(2 \ i-1)}{m_{j}} \left( \ \log { \left( 1-F_{j}(t_{(m_{j}+1-ji)};\lambda , \xi , \varphi ) \right) \ } \right) ,\ \end{aligned}$$

w.r.t. the unknown parameters $\lambda$, $\xi$, and the accelerated factor $\varphi$ or by solving the following non-linear equations:

$$\begin{aligned} \frac{\partial A}{\partial \lambda }= & {} -2 \sum _{i=1}^{m_{1}} \ \left( \delta _{1}(t_{(1i)};\lambda , \xi ) - \frac{(2 \ i-1)}{m_{1}} \ \frac{\delta _{1}(t_{(m_{1}+1-1i)};\lambda , \xi )}{ e^{\lambda \left( \ 1-e^{t_{(m_{1}+1-1i)}^{\xi }} \right) \ }} \right) \ \\ {}{} & {} \, - 2 \sum _{i=1}^{m_{2}} \ \left( \delta _{2}(t_{(2i)};\lambda , \xi , \varphi ) - \frac{(2 \ i-1)}{m_{2}} \ \frac{\delta _{2}(t_{(m_{2}+1-2i)};\lambda , \xi , \varphi )}{ e^{\lambda \ \varphi \left( 1-e^{t_{(m_{2}+1-2i)}^{\xi }} \right) \ }} \right) \ =0, \\ \frac{\partial A}{\partial \xi }= & {} -2 \sum _{i=1}^{m_{1}} \ \left( \delta _{3}(t_{(1i)};\lambda , \xi , ) - \frac{(2 \ i-1)}{m_{1}} \ \frac{\delta _{3}(t_{(m_{1}+1-1i)};\lambda , \xi )}{ e^{\lambda \left( 1-e^{t_{(m_{1}+1-1i)}^{\xi }} \right) \ }} \right) \ \\ {}{} & {} \, - 2 \sum _{i=1}^{m_{2}} \ \left( \ \delta _{4}(t_{(2i)};\lambda , \xi , \varphi ) - \frac{(2 \ i-1)}{m_{2}} \ \frac{\delta _{4}(t_{(m_{2}+1-2i)};\lambda , \xi , \varphi )}{ e^{\lambda \ \varphi \left( 1-e^{t_{(m_{2}+1-2i)}^{\xi }} \right) \ }} \right) \ =0, \end{aligned}$$

and

$$\begin{aligned} \frac{\partial A}{\partial \varphi }=- 2 \sum _{i=1}^{m_{2}} \ \left( \delta _{5}(t_{(2i)};\lambda , \xi , \varphi ) - \frac{(2 \ i-1)}{m_{2}} \ \frac{\delta _{5}(t_{(m_{2}+1-2i)};\lambda , \xi , \varphi )}{ e^{\lambda \ \varphi \left( 1-e^{t_{(m_{2}+1-2i)}^{\xi }} \right) \ }} \right) \ =0, \end{aligned}$$

where $\delta _{1}(t_{(1i)};\lambda , \xi )$, $\delta _{2}(t_{(2i)};\lambda , \xi , \varphi )$, $\delta _{3}(t_{(1i)};\lambda , \xi )$, $\delta _{4}(t_{(2i)};\lambda , \xi , \varphi )$ and $\delta _{5}(t_{(2i)};\lambda , \xi , \varphi )$ are given by (15), (16), (17), (18) and (19), respectively.

### Percentile estimation

Kao^32^ was the one who first proposed the PE, which has been used to estimate the unknown parameters of Weibull distribution. In order to apply this technique in this subsection to obtain the PE of Chen distribution using CPALT, the following equation needs to be minimized

$$\begin{aligned} P=\sum _{j=1}^2 \ \sum _{i=1}^{m_{j}} \left( \ t_{(ji)}-t_{P_{ji}} \right) ^2 \ \end{aligned}$$

where

$$\begin{aligned} t_{P_{1i}}= \Bigg ( \log { \Bigg ( 1- \frac{ \log { \Bigg ( 1-P_{1i}} \Bigg ) }{ \lambda }} \Bigg ) \Bigg )^{\frac{1}{\xi }} \quad t_{P_{2i}}= \Bigg (\log { \Bigg (1- \frac{ \log { \Bigg ( 1-P_{2i}} \Bigg ) }{ \lambda \varphi }} \Bigg ) \Bigg )^{\frac{1}{\xi }}, \quad P_{ji}= \frac{i}{m_{j}+1}. \end{aligned}$$

w.r.t. the unknown parameters $\lambda$, $\xi$, and the accelerated factor $\varphi$ or by solving the following non-linear equations:

$$\begin{aligned} \frac{\partial P}{\partial \lambda }= & {} -2 \sum _{i=1}^{m_{1}} \frac{ \Big (t_{(1i)}-t_{P_{1i}} \Big )\ \log {(1-P_{1i})} \ \Bigg (\log { \Bigg ( 1- \frac{ \log { \Big (\ 1-P_{1i}} \Big )\ }{ \lambda }} \Bigg )\ \Bigg )^{\frac{1}{\xi }-1} \ }{ \lambda ^2 \ \xi \left( 1-\frac{\log \left( 1-P_{1i}\right) }{\lambda }\right) } \\ {}{} & {} \,- 2 \sum _{i=1}^{m_{2}} \frac{ \Big (\ t_{(2i)}-t_{P_{2i}} \Big )\ \log {(1-P_{2i})} \ \Bigg ( \log { \Bigg (\ 1- \frac{ \log { \Big (1-P_{2i}} \Big )\ }{ \lambda \ \varphi }} \Bigg )\ \Bigg )^{\frac{1}{\xi }-1} \ }{ \lambda ^2 \ \xi \ \varphi \left( 1-\frac{\log \left( 1-P_{2i}\right) }{\lambda \ \varphi }\right) }=0, \\ \frac{\partial P}{\partial \xi }= & {} -2 \sum _{i=1}^{m_{1}} \frac{ \Big (t_{(1i)}-t_{P_{1i}} \Big )\ \log { \Bigg (\log { \Bigg (\ 1- \frac{ \log { \Big (1-P_{1i}} \Big )\ }{ \lambda }} \Bigg )\ \Bigg )\ } \ \Bigg (\log { \Bigg (1- \frac{ \log { \Big (1-P_{1i}} \Big )\ }{ \lambda }} \Bigg )\ \Bigg )^{\frac{1}{\xi }} \ }{ \xi ^2 } \\ {}{} & {} \, - 2 \sum _{i=1}^{m_{2}} \frac{ \Big (\ t_{(2i)}-t_{P_{2i}} \Big )\ \log {\Bigg (\log { \Bigg (1- \frac{ \log { \Big (1-P_{2i}} \Big )\ }{ \lambda \ \varphi }} \Bigg )\ \Bigg )\ } \ \Bigg (\log { \Bigg (1- \frac{ \log { \Big (1-P_{2i}} \Big )\ }{ \lambda \ \varphi }} \Bigg )\ \Bigg )^{\frac{1}{\xi }} \ }{ \xi ^2}=0, \end{aligned}$$

and

$$\begin{aligned} \frac{\partial P}{\partial \varphi }=- 2 \sum _{i=1}^{m_{2}} \frac{ \Big (t_{(2i)}-t_{P_{2i}} \Big )\ \log {(1-P_{2i})} \ \Bigg (\log { \Bigg (1- \frac{ \log { \Big (\ 1-P_{2i}} \Big )\ }{ \lambda \ \varphi }} \Bigg )\ \Bigg )^{\frac{1}{\xi }-1} \ }{ \lambda \ \xi \ \varphi ^2 \left( 1-\frac{\log \left( 1-P_{2i}\right) }{\lambda \ \varphi }\right) }=0, \end{aligned}$$

### Maximum product of spacing estimation

The method of MPSE developed by^33,34^ is applied in this subsection to estimate the Chen distribution using CPALT under complete data. To obtain the MPSE of Chen distribution under CPALT, the following function

20 $$\begin{aligned} M=\sum _{j=1}^2 \ \sum _{i=1}^{m_{j}+1} \frac{ \log {D_{ji}}}{m_{j}+1}, \end{aligned}$$

needs to maximize w.r.t. the unknown parameters $\lambda$, $\xi$, and the accelerated factor $\varphi$, where $D_{ji}$ is the uniform spacings of a random sample from the Chen distribution under CPALT and defined by

$$\begin{aligned} D_{ji}=F_{j}(t_{(ji)};\lambda , \xi , \varphi )-F_{j}(t_{(ji-1)};\lambda , \xi , \varphi ), \quad F_{j}(t_{(j0)};\lambda , \xi , \varphi )=0, \quad F_{j}(t_{(jm_{j}+1)};\lambda , \xi , \varphi )=1. \end{aligned}$$

Upon using Eqs. (2) and (7) into Eq. (20), one can show that

$$\begin{aligned} M= & {} \frac{ 1}{m_{1}+1} \ \Biggr [\ \sum _{i=2}^{m_{1}} \log { \Bigg (e^{\lambda \Big (1-e^{t_{(1i-1)}^{\xi }} \Big )\ }- e^{\lambda \Big (1-e^{t_{(1i)}^{\xi }} \Big )\ } \Bigg )\ }\\{} & {} \, + \log \Bigg (1- e^{\lambda \Big (1-e^{t_{11}^{\xi }} \Big )\ } \Bigg )\ + \log { e^{\lambda \Big (1-e^{t_{1m_{1}}^{\xi }} \Big )\ }} \Biggr ]\\ {}{} & {} \, + \frac{ 1}{m_{2}+1} \ \Biggr [\ \sum _{i=2}^{m_{2}} \log { \Bigg (e^{\lambda \ \varphi \Big (\ 1-e^{t_{(2i-1)}^{\xi }} \Big )\ }- e^{\lambda \ \varphi \Big (\ 1-e^{t_{(2i)}^{\xi }} \Big )\ } \Bigg )\ }\\ {}{} & {} \, + \log \Bigg (\ 1- e^{\lambda \ \varphi \Big (1-e^{t_{21}^{\xi }} \Big )\ } \Bigg )\ + \log { e^{\lambda \ \varphi \Big (1-e^{t_{2m_{2}}^{\xi }} \Big )\ }} \Biggr ].\ \end{aligned}$$

So, the MPSE of Chen distribution using CPALT can be obtained also by solving the following non-linear equations $\frac{\partial M}{\partial \lambda }=0$, $\frac{\partial M}{\partial \xi }=0$, and $\frac{\partial M}{\partial \varphi }=0$.

## Numerical computations

To illustrate the computation of methods presented in the previous section, two real life data sets are presented.

### Data set 1: ordered times to failure

The data presented in^35^ expressing the required failure times for ten steel samples under the influence of four stress levels are used in this subsection. Accordingly, only two levels of stress, 0.87 and 0.99 ($10^6$ psi), are used as the use condition and the accelerated condition after being modified to meet the problem being examined, see Table 1.

**Table 1:** The ordered times to failure under two stress levels.

Stress ($10^6$ psi)	Ordered lives
Use condition (0.87)	1.679, 2.20, 2.519 , 3.009 , 3.909 , 4.70, 7.53, 14.70, 27.8, 37.4
Accelerated condition (0.99)	0.80, 1.00, 1.37, 2.25, 2.95, 3.70, 6.07, 6.65, 7.05, 7.37.

**Figure 1:**
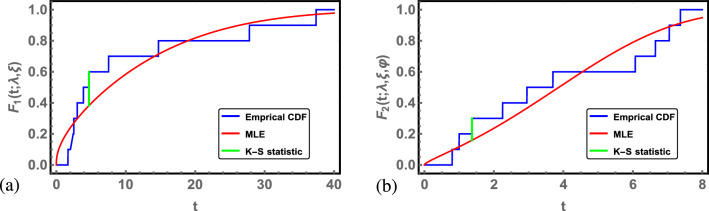
(**a**) The fitted CDF of Chen distribution under use condition for ordered times to failure data. (**b**) The fitted CDF of Chen distribution under accelerated condition for ordered times to failure data.

First, the MLE is used under complete data to check the validity of the Chen D to fit the data set for use and accelerated conditions. The Kolmogorov–Smirnov (K–S) distance and the corresponding p value are obtained for use and accelerated conditions. The results are summarized in Table 2. From Table 2, the Chen distribution provides a good fit to the data under use and accelerated conditions. Figure 1 also displays the empirical CDF and the fitted CDF of the Chen distribution using MLE in the use and accelerated conditions.

**Table 2:** The ML estimates of parameters, the K–S values and the associated p values under use and accelerated conditions.

	Estimates	*K*–*S*	p value
Use condition (0.87)	$\lambda =0.1059$, $\xi =0.3481$	0.2175	0.7316
Accelerated condition (0.99)	$\lambda =0.0562$, $\xi =0.6340$, $\varphi =1.2893$	0.1410	0.9886

The estimation methods, which are given in “Maximum likelihood estimation” to “Maximum product of spacing estimation”, are used to obtain the estimates of the unknown parameters and the accelerated factor under CPALT using the ordered times to failure data. The estimates based on real data sets under different methods of estimation are tabulated in Table 3.

**Table 3:** The different methods of estimations for $(\lambda ,\mu ,\theta ,\gamma )$ using real data.

Estimates	MLE	LSE	WLSE	CVME	ADE	RADE	QE	MPSE
${\hat{\lambda }}$	0.0642	0.0908	0.0719	0.0744	0.0970	0.1061	0.1787	0.0841
${\hat{\xi }}$	0.3971	0.4455	0.50002	0.5070	0.3665	0.3474	0.27004	0.3572
${\hat{\varphi }}$	3.3623	1.6357	1.7469	1.6372	1.9911	2.1199	2.3421	2.7257

### Data set 2: oil breakdown times of insulating fluid

The data set from^36^ that details the insulating fluid’s oil breakdown times under high test voltages are considered in this subsection after being modified to meet the problem being examined. Accordingly, only two levels of stress, 30 and 32 kV, are used as the use condition and the accelerated condition, see Table 4.

**Table 4:** Oil breakdown times of insulating fluid.

Levels stress (kv)	Breakdown times (min)
Use condition (30)	7.74, 17.05, 20.46, 21.02, 22.66, 43.40, 47.30, 139.07, 144.12, 175.88, 194.90
Accelerated condition (32)	0.27, 0.40, 0.69, 0.79, 2.75, 3.91, 9.88, 13.95, 15.93, 27.80, 53.24, 82.85, 89.29,
	100.58, 215.10

**Figure 2:**
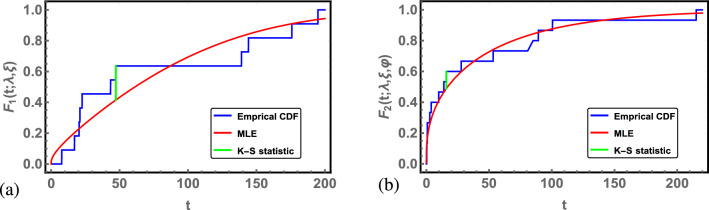
(**a**) The fitted CDF of Chen distribution under use condition for oil breakdown times of insulating fluid. (**b**) The fitted CDF of Chen distribution under accelerated condition for oil breakdown times of insulating fluid data.

First, the MLE is used under complete data to check the validity of the Chen distribution to fit the data set for use and accelerated conditions. The Kolmogorov–Smirnov (K–S) distance and the corresponding p value are obtained for use and accelerated conditions. The results are summarized in Table 5. From Table 5, the Chen distribution provides a good fit to the data under use and accelerated conditions. The empirical CDF and the fitted CDF of the Chen distribution using MLE under use and accelerated conditions are also shown in Fig. 2.

**Table 5:** The ML estimates of parameters, the K–S values and the associated p values under use and accelerated temperatures.

	Estimates	*K*–*S*	p value
Use condition	$\lambda =0.0251$, $\xi =0.2941$	0.2208	0.6568
Accelerated condition	$\lambda =0.0481$, $\xi =0.2340$, $\varphi =2.4249$	0.1108	0.9928

The estimation methods, which are given in “Maximum likelihood estimation” to “Maximum product of spacing estimation”, are used to obtain the estimates of the unknown parameters and the accelerated factor under CPALT using the oil breakdown times of insulating fluid data. The estimates based on real data sets under different methods of estimation are tabulated in Table 6.

**Table 6:** The different methods of estimations for $(\lambda ,\mu ,\theta ,\gamma )$ using real data.

Estimates	MLE	LSE	WLSE	CVME	ADE	RADE	QE	MPSE
${\hat{\lambda }}$	0.0491	0.0639	0.0549	0.0541	0.0548	0.0618	0.0577	0.0608
${\hat{\xi }}$	0.2578	0.2282	0.2392	0.2428	0.2420	0.2374	0.2441	0.2385
${\hat{\varphi }}$	1.7208	1.9772	2.0322	2.1235	1.9967	1.7793	1.4484	1.6282

## Simulation study

The principal reason for this section is to compare the estimators of the parameters by utilizing MSE and AAB. For varying values of $m_{1}$ and $m_{2}$ (number of two samples for use and accelerated conditions), a large number $N=10{,}000$ of complete samples are generated from Chen distribution under use and accelerated conditions. Take the true values of $\lambda$, $\xi$, and $\varphi$ as $(\lambda ,\xi ,\varphi )=(1.5,1.5,2)$, $(\lambda ,\xi ,\varphi )=(1.5,2,1.5)$, $(\lambda ,\xi ,\varphi )=(1.5,2,2)$ $(\lambda ,\xi ,\varphi )=(2,4,3)$, $(\lambda ,\xi ,\varphi )=(2,3,4)$, and $(\lambda ,\xi ,\varphi )=(2,3,3)$. To carry out the numerical study, the following steps are required:

1. Generate two independent random samples of sizes $m_{1}$ and $m_{2}$ from Uniform (0,1) distribution using RandomReal[] in mathematica $(U_{j1}, U_{j2}, \ldots , U_{jm_{j}}), j=1,2$. With different choice of $m_{1}$, $m_{2}$, and different values of the parameters and accelerated factor, the two complete samples are generated from the inverse CDFs $F_{1}(t)$ and $F_{2}(t)$ for use and accelerated conditions respectively as follow: $t_{1i}=\Bigg (\ \log { \Bigg (\ 1- \frac{ \log { \Big (\ 1-U_{1i}} \Big )\ }{ \lambda }} \Bigg )\ \Bigg )^{\frac{1}{\xi }}$ and $t_{2i}= \Bigg (\ \log { \Bigg (\ 1- \frac{ \log { \Big (\ 1-U_{2i}} \Big )\ }{ \lambda \ \varphi }} \Bigg )\ \Bigg )^{\frac{1}{\xi }}$, where $i=1,2,3,\ldots ,m_{j}$
2. Across using the results obtained in “Maximum likelihood estimation” to “Maximum product of spacing estimation”, the different estimates of the unknown parameters and accelerated factor are calculated. using the package FindRoot[] in Mathematica or using the Newton–Raphson algorithm.
3. Repeat Steps $1-2$, $N=10{,}000$ times.
4. Calculate the AEs, MSEs and AABs of the unknown parameters and accelerated factor from $AE=\frac{1}{N} \ \sum _{l=1}^{N}({\hat{\Theta }})$, $MSE=\frac{1}{N} \ \sum _{l=1}^{N}({\hat{\Theta }}-\Theta )^2$, and $AAB=\frac{1}{N} \ \sum _{l=1}^{N}|{\hat{\Theta }}-\Theta |$. where, ${\hat{\Theta }}$ is the parameter estimation for $\Theta$.

The results obtained from the numerical comparison study between different methods based on MSEs and AABs for all estimates are presented in Tables 7, 8, 9, 10, 11 and 12. These tables amply demonstrate that:

- It is clear from Tables 7, 8, 9, 10, 11 and 12 that with an increase in $m_{1}$, $m_{2}$, the MSEs and AABs decrease for all estimates as expected.
- It is clear also that MLE improves for the better in terms of small values of MSE and AAB and becomes one of the best estimates for large sample in relation to the parameter $\lambda$.
- We also find that the MPSE outperforms alternative techniques in most cases for parameter $\lambda$.
- For the parameter $\xi$, we find that MLE is the best estimate based on the lowest values of MSE and AAB.
- As for the parameter $\varphi$, we find that MPSE is the best estimate, followed by MLE according to MSE and AAB.
- Taking MSE and AAB into consideration, the MPSE technique outperforms alternative techniques in most cases.
- In view of the results of the simulation study, we recommend the use of MPSE, MLE, PE and ADE to estimate CPALT under the complete data when taking MSE and AAB into consideration.

**Table 7:** The average estimators, the mean square error, and the absolute average bias of different methods of estimations (1.5,1.5,2).

$(m_{1},m_{2})$	MLE	LSE	WLSE	CVME	ADE	RTADE	PE	MPSE
(20, 20)								
$$\lambda $$								
AE	1.7397	1.5840	1.6186	1.7623	1.6146	1.6429	1.5293	1.4656
MSE	0.2323	0.1889	0.1891	0.3453	0.1567	0.1760	0.1162	0.0906
AAB	0.3320	0.2952	0.2967	0.3922	0.2820	0.2899	0.2495	0.2348
$$\xi $$								
AE	1.5866	1.4570	1.4914	1.5864	1.4834	1.5195	1.4250	1.3470
MSE	0.0597	0.0758	0.0636	0.0928	0.0521	0.0635	0.0607	0.0614
AAB	0.1853	0.2079	0.1936	0.2274	0.1803	0.1936	0.1955	0.2088
$$\varphi $$								
AE	2.1659	2.0673	2.0981	2.2010	2.0835	2.1118	2.0163	1.9418
MSE	0.4863	0.4081	0.4256	0.6189	0.4085	0.4282	0.3366	0.2772
AAB	0.5194	0.4821	0.4918	0.5820	0.4918	0.4923	0.4497	0.4197
(60, 60)								
$$\lambda $$								
AE	1.5568	1.5193	1.5338	1.5672	1.5228	1.5304	1.4825	1.4522
MSE	0.0505	0.0708	0.0574	0.0795	0.0512	0.0498	0.0406	0.0379
AAB	0.1699	0.1902	0.1802	0.2030	0.1734	0.1702	0.1591	0.1567
$$\xi $$								
AE	1.5271	1.4835	1.5035	1.5262	1.4927	1.5039	1.4612	1.4190
MSE	0.0163	0.0284	0.0199	0.0282	0.0178	0.0203	0.0191	0.0201
AAB	0.1000	0.1250	0.1114	0.1261	0.1063	0.1130	0.1118	0.1176
$$\varphi $$								
AE	2.0560	2.0275	2.0452	2.0662	2.0337	2.0399	2.0014	1.9616
MSE	0.1680	0.1943	0.1892	0.2186	0.1780	0.1737	0.1521	0.1329
AAB	0.3145	0.1943	0.3349	0.3574	0.3281	0.3202	0.3042	0.2888
(100, 100)								
$$\lambda $$								
AE	1.5280	1.5064	1.5184	1.5360	1.5094	1.5133	1.4806	1.4588
MSE	0.0274	0.0392	0.0325	0.0421	0.0298	0.0285	0.0245	0.0237
AAB	0.1281	0.1483	0.1383	0.1540	0.1345	0.1309	0.1242	0.1239
$$\xi $$								
AE	1.5186	1.4925	1.5074	1.5191	1.4983	1.5052	1.4751	1.4442
MSE	0.0095	0.0154	0.0117	0.0158	0.0108	0.0123	0.0114	0.0114
AAB	0.0769	0.0955	0.0858	0.0971	0.0827	0.0883	0.0861	0.0877
$$\varphi $$								
AE	2.0426	2.0246	2.0362	2.0471	2.0280	2.0330	2.0068	1.9782
MSE	0.0946	0.1193	0.1113	0.1281	0.1067	0.1011	0.0911	0.0801
AAB	0.2401	0.2679	0.2589	0.2759	0.2552	0.2475	0.2369	0.2256
(150, 150)								
$$\lambda $$								
AE	1.5182	1.5021	1.5122	1.5216	1.5056	1.5082	1.4846	1.4680
MSE	0.0170	0.0252	0.0203	0.0259	0.0192	0.0180	0.0159	0.0156
AAB	0.1024	0.1208	0.1116	0.1233	0.1093	0.1057	0.1008	0.1011
$$\xi $$								
AE	1.5125	1.4943	1.5056	1.5118	1.4988	1.5035	1.4812	1.4576
MSE	0.0061	0.0101	0.0075	0.0101	0.0071	0.0079	0.0073	0.0074
AAB	0.0622	0.0770	0.0685	0.0776	0.0669	0.0705	0.0686	0.0698
$$\varphi $$								
AE	2.0227	2.0115	2.0193	2.0259	2.0133	2.0164	1.9973	1.9761
MSE	0.0614	0.0776	0.0710	0.0810	0.0688	0.0649	0.0598	0.0550
AAB	0.1955	0.2198	0.2103	0.2237	0.2079	0.2018	0.1948	0.1874
(200, 200)								
$$\lambda $$								
AE	1.5141	1.5004	1.5091	1.5152	1.5036	1.5069	1.4882	1.4741
MSE	0.0130	0.0175	0.0151	0.0184	0.0145	0.0139	0.0124	0.0121
AAB	0.0897	0.1047	0.0968	0.1066	0.0954	0.0931	0.0890	0.0885
$$\xi $$								
AE	1.5097	1.4962	1.5051	1.5096	1.4994	1.5039	1.4862	1.4656
MSE	0.0046	0.0070	0.0056	0.0073	0.0054	0.0060	0.0055	0.0054
AAB	0.0536	0.0667	0.0595	0.0675	0.0584	0.0616	0.0592	0.0596
$$\varphi $$								
AE	2.0172	2.0114	2.0168	2.0222	2.0120	2.0136	1.9986	1.9801
MSE	0.0448	0.0574	0.0523	0.0594	0.0509	0.0480	0.0445	0.0411
AAB	0.1670	0.1894	0.1805	0.1921	0.1789	0.1734	0.1677	0.1619
(300, 300)								
$$\lambda $$								
AE	1.5075	1.4996	1.5053	1.5094	1.5014	1.5026	1.4889	1.4786
MSE	0.0084	0.0116	0.0098	0.0120	0.0096	0.0090	0.0082	0.0082
AAB	0.0730	0.0861	0.0789	0.0780	0.0782	0.0758	0.0730	0.0731
$$\xi $$								
AE	1.5046	1.4958	1.5020	1.5047	1.4980	1.5000	1.4870	1.4724
MSE	0.0029	0.0046	0.0036	0.0046	0.0035	0.0039	0.0037	0.0036
AAB	0.0432	0.0539	0.0477	0.0541	0.0471	0.0500	0.0488	0.0482
$$\varphi $$								
AE	2.0115	2.0065	2.0104	2.0135	2.0072	2.0082	1.9973	1.9844
MSE	0.0292	0.0378	0.0341	0.0387	0.0336	0.0313	0.0295	0.0275
AAB	0.1359	0.1543	0.1460	0.1557	0.1457	0.1408	0.1369	0.1327

**Table 8:** The average estimators, the mean square error, and the absolute average bias of different methods of estimations (1.5,2,1.5).

$(m_{1},m_{2})$	MLE	LSE	WLSE	CVME	ADE	RTADE	PE	MPSE
(20, 20)								
$$\lambda $$								
AE	1.7141	1.5717	1.6073	1.7461	1.5934	1.6174	1.5096	1.4464
MSE	0.2316	0.1970	0.2030	0.3606	0.1626	0.1754	0.1168	0.0981
AAB	0.3332	0.3062	0.3082	0.4013	0.2901	0.2951	0.2575	0.2461
$$\xi $$								
AE	2.1191	1.9468	1.9972	2.1219	1.9800	2.0221	1.8986	1.7993
MSE	0.1115	0.1358	0.1184	0.1754	0.0963	0.1147	0.0934	0.1107
AAB	0.2527	0.2810	0.2622	0.3109	0.2446	0.2614	0.2488	0.2803
$$\varphi $$								
AE	1.6479	1.5973	1.6189	1.6770	1.6106	1.6192	1.5753	1.5309
MSE	0.3353	0.2992	0.3176	0.4326	0.2977	0.2970	0.2503	0.1976
AAB	0.4166	0.3929	0.4041	0.4660	0.4017	0.3939	0.3706	0.3361
(60, 60)								
$$\lambda $$								
AE	1.5557	1.5192	1.5353	1.5676	1.5221	1.5284	1.4848	1.4512
MSE	0.0505	0.0694	0.0589	0.0781	0.0505	0.0491	0.0418	0.0382
AAB	0.1714	0.1900	0.1819	0.2040	0.1741	0.1707	0.1617	0.1576
$$\xi $$								
AE	2.0407	1.9815	2.0104	2.0401	1.9944	2.0086	1.9544	1.8958
MSE	0.0296	0.0486	0.0369	0.0998	0.0317	0.0362	0.0301	0.0350
AAB	0.1348	0.1639	0.1486	0.1685	0.1415	0.1503	0.1400	0.1545
$$\varphi $$								
AE	1.5428	1.5287	1.5376	1.5483	1.5329	1.5357	1.5193	1.4983
MSE	0.0893	0.1057	0.1022	0.1177	0.0969	0.0915	0.0863	0.0721
AAB	0.2315	0.2496	0.2462	0.2623	0.2418	0.2337	0.2289	0.2116
(100, 100)								
$$\lambda $$								
AE	1.5291	1.5060	1.5184	1.5348	1.5094	1.5136	1.4843	1.4597
MSE	0.0273	0.0388	0.0316	0.0409	0.0293	0.0281	0.0251	0.0235
AAB	0.1290	0.1486	0.1387	0.1541	0.1350	0.1316	0.1264	0.1238
$$\xi $$								
AE	2.0237	1.9867	2.0071	2.0216	1.9951	2.0044	1.9677	1.9243
MSE	0.0167	0.0284	0.0209	0.0283	0.0193	0.0215	0.0181	0.0203
AAB	0.1014	0.1284	0.1139	0.1295	0.1102	0.1153	0.1078	0.1165
$$\varphi $$								
AE	1.5219	1.5137	1.5191	1.5243	1.5155	1.5170	1.5069	1.4927
MSE	0.0499	0.0629	0.0582	0.0663	0.0564	0.0526	0.0508	0.0434
AAB	0.1752	0.1971	0.1898	0.2018	0.1875	0.1803	0.1782	0.1654
(150, 150)								
$$\lambda $$								
AE	1.5172	1.5016	1.5115	1.5216	1.5048	1.5076	1.4862	1.4668
MSE	0.0173	0.0235	0.0205	0.0257	0.0193	0.0184	0.0168	0.0160
AAB	0.1032	0.1205	0.1121	0.1238	0.1100	0.1069	0.1037	0.1021
$$\xi $$								
AE	2.0171	1.9938	2.0087	2.0177	1.9993	2.0051	1.9788	1.9435
MSE	0.0110	0.0171	0.0138	0.0182	0.0129	0.0146	0.0119	0.0131
AAB	0.0831	0.1042	0.0928	0.1057	0.0907	0.0962	0.0882	0.0936
$$\varphi $$								
AE	1.5149	1.5120	1.5150	1.5186	1.5122	1.5123	1.5057	1.4937
MSE	0.0328	0.0424	0.0388	0.0439	0.0377	0.0349	0.0345	0.0296
AAB	0.1433	0.1626	0.1554	0.1652	0.1538	0.1482	0.1471	0.1372
(200, 200)								
$$\lambda $$								
AE	1.5142	1.5032	1.5110	1.5181	1.5056	1.5073	1.4903	1.4740
MSE	0.0130	0.0175	0.0151	0.0185	0.0145	0.0137	0.0127	0.0121
AAB	0.0898	0.1045	0.0967	0.1065	0.0955	0.0927	0.0902	0.0888
$$\xi $$								
AE	2.0126	1.9962	2.0077	2.0140	2.0000	2.0034	1.9831	1.9536
MSE	0.0082	0.0130	0.0103	0.0135	0.0098	0.0108	0.0090	0.0098
AAB	0.0719	0.0913	0.0807	0.0922	0.0794	0.0823	0.0763	0.0807
$$\varphi $$								
AE	1.5116	1.5101	1.5121	1.5149	1.5099	1.5097	1.5046	1.4948
MSE	0.0240	0.0310	0.0283	0.0318	0.0277	0.0254	0.0253	0.0222
AAB	0.1223	0.1392	0.1329	0.1409	0.1317	0.1260	0.1261	0.1183
(300, 300)								
$$\lambda $$								
AE	1.5081	1.5003	1.5060	1.5102	1.5021	1.5032	1.4913	1.4792
MSE	0.0085	0.0117	0.0100	0.0122	0.0097	0.0092	0.0086	0.0083
AAB	0.0729	0.0853	0.0787	0.0863	0.0780	0.0759	0.0740	0.0731
$$\xi $$								
AE	2.0082	1.9965	2.0047	2.0083	1.9994	2.0015	1.9871	1.9651
MSE	0.0053	0.0083	0.0065	0.0084	0.0063	0.0069	0.0058	0.0062
AAB	0.0574	0.0727	0.0642	0.0731	0.0633	0.0659	0.0610	0.0638
$$\varphi $$								
AE	1.5079	1.5063	1.5079	1.5095	1.5064	1.5064	1.5027	1.4956
MSE	0.0160	0.0204	0.0185	0.0207	0.0183	0.0170	0.0169	0.0151
AAB	0.1003	0.1133	0.1080	0.1141	0.1074	0.1037	0.1034	0.0978

**Table 9:** The average estimators, the mean square error, and the absolute average bias of different methods of estimations (1.5,2,2).

$(m_{1},m_{2})$	MLE	LSE	WLSE	CVME	ADE	RTADE	PE	MPSE
(20, 20)								
$$\lambda $$								
AE	1.7220	1.5723	1.6093	1.7464	1.5999	1.6233	1.5140	1.4530
MSE	0.2311	0.1924	0.2016	0.3573	0.1637	0.1750	0.1158	0.0959
AAB	0.3325	0.3046	0.3070	0.4004	0.2912	0.2930	0.2562	0.2444
$$\xi $$								
AE	2.1154	1.9382	1.9884	2.1092	1.9758	2.0202	1.8933	1.7967
MSE	0.1080	0.1298	0.1135	0.1633	0.0934	0.1148	0.0951	0.1103
AAB	0.2506	0.2769	0.2606	0.3056	0.2435	0.2603	0.2511	0.2810
$$\varphi $$								
AE	2.1899	2.0813	2.1144	2.2213	2.1030	2.1310	2.0393	1.9605
MSE	0.5068	0.4136	0.4333	0.6370	0.4185	0.4386	0.3512	0.2822
AAB	0.5298	0.4848	0.4964	0.5910	0.4980	0.4973	0.4603	0.4230
(60, 60)								
$$\lambda $$								
AE	1.5521	1.5188	1.5324	1.5667	1.5194	1.5262	1.4809	1.4481
MSE	0.0475	0.0682	0.0566	0.0778	0.0492	0.0475	0.0397	0.0365
AAB	0.1671	0.1886	0.1800	0.2029	0.1727	0.1681	0.1582	0.1550
$$\xi $$								
AE	2.0387	1.9822	2.0096	2.0399	1.9936	2.0068	1.9534	1.8945
MSE	0.0291	0.0492	0.0362	0.0495	0.0319	0.0359	0.0311	0.0365
AAB	0.1336	0.1653	0.1488	0.1685	0.1422	0.1494	0.1427	0.1546
$$\varphi $$								
AE	2.0645	2.0361	2.0547	2.0766	2.0432	2.0465	2.0148	1.9694
MSE	0.1658	0.1958	0.1902	0.2226	0.1783	0.1693	0.1582	0.1300
AAB	0.3126	0.3372	0.3336	0.3569	0.3266	0.3165	0.3089	0.2850
(100, 100)								
$$\lambda $$								
AE	1.5271	1.5053	1.5161	1.5335	1.5073	1.5125	1.4818	1.4580
MSE	0.0274	0.0408	0.0318	0.0412	0.0296	0.0286	0.0252	0.0238
AAB	0.1286	0.1492	0.1388	0.1534	0.1351	0.1321	0.1270	0.1255
$$\xi $$								
AE	2.0242	1.9894	2.0083	2.0238	1.9967	2.0068	1.9692	1.9252
MSE	0.0168	0.0286	0.0204	0.0277	0.0189	0.0214	0.0182	0.0203
AAB	0.1019	0.1274	0.1134	0.1287	0.1099	0.1157	0.1085	0.1167
$$\varphi $$								
AE	2.0401	2.0226	2.0342	2.0453	2.0269	2.0303	2.0081	1.9761
MSE	0.0977	0.1212	0.1132	0.1298	0.1087	0.1035	0.0979	0.0830
AAB	0.2430	0.2698	0.2607	0.2776	0.2572	0.2499	0.2451	0.2285
(150, 150)								
$$\lambda $$								
AE	1.5169	1.5008	1.5111	1.5206	1.5045	1.5071	1.4853	1.4667
MSE	0.0177	0.0237	0.0208	0.0254	0.0197	0.0188	0.0170	0.0162
AAB	0.1042	0.1208	0.1129	0.1235	0.1107	0.1078	0.1042	0.1031
$$\xi $$								
AE	2.0148	1.9911	2.0061	2.0148	1.9971	2.0029	1.9758	1.9418
MSE	0.0110	0.0169	0.0136	0.0175	0.0129	0.0148	0.0124	0.0134
AAB	0.0833	0.1035	0.0926	0.1044	0.0904	0.0966	0.0895	0.0949
$$\varphi $$								
AE	2.0215	2.0116	2.0188	2.0262	2.0130	2.0156	1.9988	1.9750
MSE	0.0616	0.0779	0.0713	0.0813	0.0692	0.0657	0.0632	0.0553
AAB	0.1952	0.2200	0.2099	0.2238	0.2075	0.2021	0.1988	0.1876
(200, 200)								
$$\lambda $$								
AE	1.5143	1.5006	1.5098	1.5154	1.5044	1.5067	1.4896	1.4743
MSE	0.0131	0.0177	0.0153	0.0186	0.0147	0.0140	0.0127	0.0122
AAB	0.0902	0.1048	0.0973	0.1066	0.0959	0.0933	0.0902	0.0890
$$\xi $$								
AE	2.0125	1.9929	2.0057	2.0106	1.9982	2.0934	1.9817	1.9538
MSE	0.0081	0.0126	0.0101	0.0129	0.0097	0.0109	0.0092	0.0097
AAB	0.0715	0.0893	0.0793	0.0899	0.0778	0.0825	0.0765	0.0796
$$\varphi $$								
AE	2.0178	2.0090	2.0152	2.0197	2.0106	2.0129	1.9998	1.9806
MSE	0.0449	0.0582	0.0529	0.0601	0.0516	0.0485	0.0472	0.0411
AAB	0.1669	0.1908	0.1815	0.1933	0.1797	0.1744	0.1721	0.1614
(300, 300)								
$$\lambda $$								
AE	1.5085	1.5002	1.5063	1.5100	1.5024	1.5035	1.4915	1.4797
MSE	0.0084	0.0116	0.0099	0.0121	0.0096	0.0090	0.0084	0.0081
AAB	0.0726	0.0856	0.0787	0.0866	0.0777	0.0752	0.0733	0.0725
$$\xi $$								
AE	2.0089	1.9960	2.0048	2.0078	1.9994	2.0022	1.9872	1.9659
MSE	0.0055	0.0084	0.0066	0.0086	0.0064	0.0071	0.0061	0.0063
AAB	0.0592	0.0731	0.0650	0.0737	0.0641	0.0624	0.0628	0.0646
$$\varphi $$								
AE	2.0143	2.0076	2.0120	2.0147	2.0087	2.0100	2.0010	1.9871
MSE	0.0302	0.0394	0.0355	0.0402	0.0350	0.0326	0.0322	0.0283
AAB	0.1378	0.1579	0.1500	0.1593	0.1490	0.1437	0.1431	0.1343

**Table 10:** The average estimators, the mean square error, and the absolute average bias of different methods of estimations (2,4,3).

$(m_{1},m_{2})$	MLE	LSE	WLSE	CVME	ADE	RTADE	PE	MPSE
(20, 20)								
$$\lambda $$								
AE	2.3111	2.0802	2.1489	2.3434	2.1124	2.1564	1.9602	1.8757
MSE	0.5026	0.4311	0.4607	0.8044	0.3404	0.3926	0.2499	0.2121
AAB	0.4821	0.4407	0.4483	0.5722	0.4140	0.4261	0.3800	0.3652
$$\xi $$								
AE	4.2052	3.8785	3.9832	4.1930	3.9366	4.0209	3.7287	3.5812
MSE	0.4127	0.5298	0.5036	0.6159	0.3640	0.4481	0.4048	0.4506
AAB	0.4905	0.5590	0.5327	0.5942	0.4817	0.5185	0.5228	0.5664
$$\varphi $$								
AE	3.4473	3.2000	3.2847	3.5155	3.2459	3.3116	3.0930	2.9206
MSE	1.6668	1.2747	1.4229	2.1635	1.2709	1.4075	1.1451	0.7897
AAB	0.9101	0.8021	0.8359	1.0136	0.8186	0.8349	0.7903	0.6943
(60, 60)								
$$\lambda $$								
AE	2.0857	2.0396	2.0686	2.1126	2.0328	2.0441	1.9604	1.9105
MSE	0.1047	0.1559	0.1617	0.1747	0.1049	0.1036	0.0910	0.0797
AAB	0.2425	0.2781	0.2678	0.2976	0.2474	0.2449	0.2392	0.2293
$$\xi $$								
AE	4.0723	3.9836	4.0312	4.0889	3.9868	4.0122	3.8785	3.7886
MSE	0.1174	0.2276	0.1992	0.2170	0.1277	0.1475	0.1323	0.1429
AAB	0.2672	0.3391	0.3071	0.3416	0.2828	0.3024	0.2942	0.3123
$$\varphi $$								
AE	3.1299	3.0802	3.1051	3.1642	3.0758	3.0928	3.0004	2.9175
MSE	0.4269	0.5071	0.4864	0.5914	0.4408	0.4441	0.4271	0.3210
AAB	0.4935	0.5308	0.5189	0.5683	0.5070	0.5023	0.5052	0.4492
(100, 100)								
$$\lambda $$								
AE	2.0474	2.0140	2.0313	2.0577	2.0142	2.0223	1.9654	1.9303
MSE	0.0542	0.0827	0.0716	0.0876	0.0578	0.0565	0.0533	0.0472
AAB	0.1798	0.2098	0.1950	0.2178	0.1879	0.1852	0.1837	0.1756
$$\xi $$								
AE	4.0473	3.9858	4.0174	4.0509	3.9936	4.0111	3.9193	3.8527
MSE	0.0686	0.1273	0.0906	0.1229	0.0767	0.0883	0.0786	0.0823
AAB	0.2064	0.2600	0.2295	0.2609	0.2209	0.2362	0.2261	0.2350
$$\varphi $$								
AE	3.0722	3.0421	3.0598	3.0923	3.0421	3.0510	2.9919	2.9300
MSE	0.2436	0.3072	0.2842	0.3367	0.2695	0.2604	0.2723	0.2350
AAB	0.3824	0.4242	0.4097	0.4408	0.4033	0.3953	0.4089	0.3607
(150, 150)								
$$\lambda $$								
AE	2.0284	2.0036	2.0186	2.0343	2.0073	2.0130	1.9712	1.9434
MSE	0.0352	0.0513	0.0426	0.0542	0.0397	0.0382	0.0374	0.0328
AAB	0.1462	0.1733	0.1596	0.1775	0.1565	0.1536	0.1543	0.1470
$$\xi $$								
AE	4.0293	3.9834	4.0120	4.0290	3.9938	4.0073	3.9386	3.8861
MSE	0.0436	0.0718	0.0551	0.0713	0.0509	0.0570	0.0526	0.0529
AAB	0.1660	0.2065	0.1836	0.2071	0.1798	0.1910	0.1841	0.1873
$$\varphi $$								
AE	3.0456	3.0199	3.0375	3.0529	3.0237	3.0319	2.8971	2.9419
MSE	0.1507	0.1941	0.1777	0.2054	0.1708	0.1633	0.1765	0.1339
AAB	0.3028	0.3445	0.3291	0.3526	0.3249	0.3169	0.3318	0.2924
(200, 200)								
$$\lambda $$								
AE	2.0232	2.0042	2.0164	2.0276	2.0073	2.0114	1.9784	1.9553
MSE	0.0261	0.0373	0.0310	0.0393	0.0296	0.0285	0.0281	0.0247
AAB	0.1268	0.1506	0.1381	0.1537	0.1357	0.1329	0.1339	0.1274
$$\xi $$								
AE	4.0227	3.9880	4.0105	4.0226	3.9959	4.0059	3.9514	3.9079
MSE	0.0323	0.0502	0.0390	0.0509	0.0374	0.0431	0.0384	0.0387
AAB	0.1421	0.1753	0.1565	0.1767	0.1533	0.1644	0.1568	0.1602
$$\varphi $$								
AE	3.0279	3.0087	3.0223	3.0335	3.0115	3.0180	2.9815	2.9454
MSE	0.1107	0.1441	0.1302	0.1502	0.1267	0.1211	0.1328	0.1021
AAB	0.2631	0.2290	0.2844	0.3038	0.2820	0.2749	0.2901	0.2573
(300, 300)								
$$\lambda $$								
AE	2.0150	2.0025	2.0119	2.0183	2.0053	2.0088	1.9841	1.9658
MSE	0.0172	0.0242	0.0204	0.0252	0.0197	0.0192	0.0189	0.0168
AAB	0.1038	0.1232	0.1130	0.1251	0.1117	0.1101	0.1101	0.1045
$$\xi $$								
AE	4.0173	3.9948	4.0114	4.0181	4.0005	4.0088	3.9679	3.9334
MSE	0.0214	0.0333	0.0262	0.0341	0.0255	0.0292	0.0255	0.0247
AAB	0.1162	0.1447	0.1286	0.1458	0.1271	0.1353	0.1283	0.1273
$$\varphi $$								
AE	3.0227	3.0073	3.0185	3.0239	3.0106	3.0166	2.9890	2.9624
MSE	0.0743	0.0963	0.0863	0.0990	0.0846	0.0816	0.0884	0.0698
AAB	0.2155	0.2455	0.2325	0.2483	0.2306	0.2263	0.2359	0.2116

**Table 11:** The average estimators, the mean square error, and the absolute average bias of different methods of estimations (2,3,4).

$(m_{1},m_{2})$	MLE	LSE	WLSE	CVME	ADE	RTADE	PE	MPSE
(20, 20)								
$$\lambda $$								
AE	2.3110	2.0693	2.1401	2.3504	2.1221	2.1609	1.9745	1.8777
MSE	0.4764	0.3770	0.4129	0.7541	0.3241	0.3662	0.2276	0.2007
AAB	0.4724	0.4266	0.4359	0.5689	0.4039	0.4139	0.3607	0.3544
$$\xi $$								
AE	3.1346	2.8798	2.9542	3.1261	2.9389	3.0003	2.7969	2.6724
MSE	0.2091	0.2636	0.2420	0.3149	0.1909	0.2407	0.2166	0.2494
AAB	0.3536	0.4067	0.3848	0.4312	0.3519	0.3821	0.3840	0.4228
$$\varphi $$								
AE	4.5526	4.1097	4.2233	4.5833	4.2010	4.3246	3.9674	3.7287
MSE	2.4319	1.6558	1.7853	2.8868	1.6725	1.9952	1.5312	1.1561
AAB	1.1460	0.9879	1.0165	1.2484	0.9950	1.0439	0.9664	0.8836
(60, 60)								
$$\lambda $$								
AE	2.0770	2.0154	2.0506	2.0917	2.0222	2.0336	1.9562	1.9040
MSE	0.0985	0.1258	0.1382	0.1500	0.0989	0.0981	0.0821	0.0777
AAB	0.2368	0.2644	0.2559	0.2836	0.2421	0.2392	0.2284	0.2271
$$\xi $$								
AE	3.0526	2.9768	3.0176	3.0571	2.9881	3.0084	2.9181	2.8419
MSE	0.0667	0.1050	0.1059	0.1080	0.0718	0.0854	0.0751	0.0811
AAB	0.2026	0.2450	0.2288	0.2501	0.2125	0.2293	0.2210	0.2345
$$\varphi $$								
AE	4.2205	4.1383	4.1872	4.2768	4.1331	4.1686	4.0165	3.8667
MSE	0.8369	0.9741	0.9726	1.1655	0.8453	0.8817	0.7937	0.6027
AAB	0.6898	0.7302	0.7280	0.7900	0.6995	0.7022	0.6864	0.6206
(100, 100)								
$$\lambda $$								
AE	2.0406	2.0074	2.0258	2.0512	2.0089	2.0154	1.9639	1.9251
MSE	0.0535	0.0775	0.0673	0.0843	0.0577	0.0559	0.0498	0.0479
AAB	0.1789	0.2088	0.1939	0.2163	0.1879	0.1841	0.1786	0.1783
$$\xi $$								
AE	3.0310	2.9851	3.0104	3.0338	2.9915	3.0037	2.9434	2.8869
MSE	0.0372	0.0639	0.0504	0.0633	0.0418	0.0490	0.0440	0.046
AAB	0.1521	0.1892	0.1693	0.1899	0.1630	0.1748	0.1683	0.1753
$$\varphi $$								
AE	4.1222	4.0701	4.1034	4.1527	4.0673	4.0879	3.9911	3.8862
MSE	0.4572	0.5753	0.5454	0.6366	0.4922	0.4913	0.4815	0.3750
AAB	0.5192	0.5745	0.5572	0.5995	0.5430	0.5384	0.5419	0.4875
(150, 150)								
$$\lambda $$								
AE	2.0290	2.0046	2.0192	2.0351	2.0079	2.0134	1.9749	1.9448
MSE	0.0340	0.0496	0.0411	0.0527	0.0380	0.0369	0.0331	0.0317
AAB	0.1438	0.1698	0.1563	0.1745	0.1531	0.1507	0.1450	0.1439
$$\xi $$								
AE	3.0236	2.9907	3.0107	3.0246	2.9975	3.0067	2.9618	2.9173
MSE	0.0247	0.0408	0.0306	0.0412	0.0280	0.0325	0.0284	0.0295
AAB	0.1243	0.1534	0.1368	0.1552	0.1337	0.1433	0.1353	0.1397
$$\varphi $$								
AE	4.0766	4.0392	4.0637	4.0937	4.0411	4.0550	3.9843	3.9045
MSE	0.2931	0.3831	0.3454	0.4102	0.3274	0.3241	0.3214	0.2571
AAB	0.4208	0.4790	0.4559	0.4927	0.4480	0.4452	0.4464	0.4064
(200, 200)								
$$\lambda $$								
AE	2.0194	2.0010	2.0126	2.0235	2.0036	2.0080	1.9779	1.9521
MSE	0.0261	0.0379	0.0313	0.0393	0.0293	0.0285	0.0261	0.0251
AAB	0.1270	0.1508	0.1385	0.1533	0.1363	0.1333	0.1298	0.1284
$$\xi $$								
AE	3.0160	2.9913	3.0076	3.0164	2.9965	3.0035	2.9688	2.9308
MSE	0.0178	0.0299	0.0226	0.0295	0.0207	0.0238	0.0211	0.0215
AAB	0.1055	0.1326	0.1169	0.1327	0.1148	0.1225	0.1170	0.1192
$$\varphi $$								
AE	4.0538	4.0268	4.0473	4.0672	4.0282	4.0371	3.9845	3.9168
MSE	0.2158	0.2881	0.2575	0.3005	0.2445	0.2396	0.2429	0.1966
AAB	0.3657	0.4189	0.3958	0.4266	0.3906	0.3854	0.3904	0.3557
(300, 300)								
$$\lambda $$								
AE	2.0135	2.0002	2.0088	2.0158	2.0026	2.0054	1.9840	1.9648
MSE	0.0164	0.0246	0.0195	0.0255	0.0189	0.0183	0.0168	0.0162
AAB	0.1011	0.1219	0.1101	0.1234	0.1088	0.1069	0.1036	0.1027
$$\xi $$								
AE	3.0111	2.9935	3.0050	3.0107	2.9973	3.0023	2.9771	2.9491
MSE	0.0117	0.0194	0.0139	0.0196	0.0136	0.0157	0.0139	0.0137
AAB	0.0856	0.1069	0.0936	0.1073	0.0928	0.0998	0.0943	0.0945
$$\varphi $$								
AE	4.0351	4.0132	4.0279	4.0405	4.0155	4.0232	3.9838	3.9356
MSE	0.1453	0.1925	0.1679	0.1979	0.1646	0.1604	0.1664	0.1366
AAB	0.3010	0.3429	0.3217	0.3469	0.3197	0.3160	0.3231	0.2966

**Table 12:** The average estimators, the mean square error, and the absolute average bias of different methods of estimations (2,3,3).

$(m_{1},m_{2})$	MLE	LSE	WLSE	CVME	ADE	RTADE	PE	MPSE
(20, 20)								
$$\lambda $$								
AE	2.3294	2.0983	2.1612	2.3710	2.1320	2.1754	1.9844	1.8887
MSE	0.5100	0.4354	0.4736	0.8326	0.3414	0.3888	0.2372	0.2076
AAB	0.4878	0.4437	0.4503	0.5862	0.4142	0.4274	0.3687	0.3615
$$\xi $$								
AE	3.1482	2.9081	2.9787	3.1464	2.9481	3.0132	2.8051	2.6803
MSE	0.2304	0.3140	0.2923	0.3675	0.2061	0.2540	0.2220	0.2570
AAB	0.3680	0.4262	0.3995	0.4523	0.3632	0.3901	0.3883	0.4272
$$\varphi $$								
AE	3.4061	3.1646	3.2365	3.4680	3.1987	3.2745	3.0465	2.8884
MSE	1.5825	1.2324	1.3175	2.0688	1.1999	1.3680	1.0278	0.7704
AAB	0.8911	0.7973	0.8228	0.9998	0.8059	0.8313	0.7595	0.6927
(60, 60)								
$$\lambda $$								
AE	2.0851	2.0356	2.0620	2.1116	2.0308	2.0435	1.9641	1.9098
MSE	0.1015	0.1582	0.1636	0.1798	0.1017	0.1015	0.0835	0.0776
AAB	0.2396	0.2750	0.2629	0.2951	0.2449	0.2423	0.2292	0.2266
$$\xi $$								
AE	3.0491	2.9817	3.0156	3.0625	2.9847	3.0048	2.0147	2.8364
MSE	0.0654	0.1334	0.1171	0.1310	0.0713	0.0830	0.0724	0.0819
AAB	0.2006	0.2561	0.2310	0.2593	0.2135	0.2286	0.2178	0.2368
$$\varphi $$								
AE	3.1168	3.0721	3.0982	3.1569	3.0666	3.0816	2.9985	2.9058
MSE	0.4136	0.5020	0.4879	0.5822	0.4347	0.4279	0.4055	0.3153
AAB	0.4887	0.5295	0.5203	0.5637	0.5065	0.4987	0.4951	0.4464
(100, 100)								
$$\lambda $$								
AE	2.0458	2.0195	2.0357	2.0627	2.0138	2.0219	1.9686	1.9289
MSE	0.0543	0.1032	0.0967	0.0986	0.0576	0.0574	0.0496	0.0476
AAB	0.1815	0.2154	0.1998	0.2222	0.1883	0.1880	0.1783	0.1778
$$\xi $$								
AE	3.0344	2.9949	3.0175	3.0423	2.9947	3.0081	2.9461	2.8886
MSE	0.0382	0.0921	0.0705	0.0798	0.0427	0.0503	0.0426	0.0463
AAB	0.1542	0.1982	0.1737	0.1973	0.1645	0.1779	0.1665	0.1758
$$\varphi $$								
AE	3.0671	3.0411	3.0556	3.0870	3.0352	3.0457	2.9892	2.9254
MSE	0.2351	0.3079	0.2790	0.3262	0.2596	0.2506	0.2493	0.1986
AAB	0.3761	0.4228	0.4059	0.4361	0.3989	0.3894	0.3933	0.3558
(150, 150)								
$$\lambda $$								
AE	2.0291	2.0079	2.0204	2.0388	2.0092	2.0143	1.9762	1.9441
MSE	0.0350	0.0536	0.0417	0.0572	0.0392	0.0379	0.0344	0.0326
AAB	0.1466	0.1754	0.1601	0.1802	0.1570	0.1539	0.1487	0.1467
$$\xi $$								
AE	3.0226	2.9907	3.0096	3.0248	2.9962	3.0057	2.9606	2.9153
MSE	0.0244	0.0441	0.0298	0.0441	0.0282	0.0324	0.0280	0.0296
AAB	0.1237	0.1552	0.1365	0.1563	0.1336	0.1424	0.1343	0.1397
$$\varphi $$								
AE	3.0470	3.0187	3.0355	3.0514	3.0222	3.0308	2.9898	2.9433
MSE	0.1544	0.1967	0.1772	0.2063	0.1715	0.1651	0.1677	0.1369
AAB	0.3086	0.3466	0.3306	0.3543	0.3269	0.3197	0.3250	0.2969
(200, 200)								
$$\lambda $$								
AE	2.0217	2.0018	2.0130	2.0242	2.0041	2.0092	1.9790	1.9539
MSE	0.0256	0.0407	0.0305	0.0412	0.0292	0.0281	0.0259	0.0245
AAB	0.1261	0.1524	0.1374	0.1544	0.1355	0.1327	0.1288	0.1266
$$\xi $$								
AE	3.0197	2.9939	3.0096	3.0189	2.9988	3.0070	2.9715	2.9335
MSE	0.0186	0.0330	0.0224	0.0319	0.0215	0.0247	0.0211	0.0218
AAB	0.1080	0.1349	0.1185	0.1350	0.1167	0.1247	0.1166	0.1195
$$\varphi $$								
AE	3.0350	3.0194	3.0315	3.0435	3.0209	3.0263	2.9951	2.9522
MSE	0.1103	0.1472	0.1305	0.1526	0.1266	0.1214	0.1241	0.1006
AAB	0.2619	0.3003	0.2837	0.3049	0.2808	0.2745	0.2786	0.2542
(300, 300)								
$$\lambda $$								
AE	2.0130	2.0012	2.0094	2.0170	2.0029	2.0049	1.9842	1.9640
MSE	0.0172	0.0255	0.0204	0.0265	0.0198	0.0190	0.0177	0.0169
AAB	0.1037	0.1237	0.1126	0.1253	0.1112	0.1089	0.1066	0.1052
$$\xi $$								
AE	3.0112	2.9940	3.0059	3.0114	2.9979	3.0021	2.9775	2.9483
MSE	0.0118	0.0194	0.0144	0.0198	0.0140	0.0161	0.0136	0.0139
AAB	0.0860	0.1081	0.0952	0.1086	0.0941	0.1012	0.0932	0.0952
$$\varphi $$								
AE	3.0261	3.0119	3.0226	3.0284	3.0149	3.0191	2.9955	2.9656
MSE	0.0758	0.0974	0.0877	0.1002	0.0859	0.0831	0.0845	0.0708
AAB	0.2171	0.2464	0.2337	0.2491	0.2320	0.2277	0.2311	0.2124

## Conclusion

In this paper, the problem of various techniques of estimations under complete sample in CPALT has been studied. Eight methodologies of classical estimation, namely, MLEs, LSEs, WLSEs, CVMEs, ADEs, RTADEs, PEs, and MPSEs, have been considered to estimate the unknown parameters and the accelerated factor of Chen distribution under CPALT. The proposed methodologies were demonstrated using two real data sets, demonstrating their applicability as they can be applied to address several engineering-related problems. Additionally, in order to compare these methodologies with various sample sizes and various sets of the unknown parameters, and the accelerated factor, a comprehensive simulation analysis has been carried out. The AEs, MSEs, and AAB under complete data using CPALT have been calculated. According to the MSEs and AABs values computed from the simulation study, the MPSE is the most effective methodology among the alternatives in most cases for all parameters. Based on the results of the simulation study, it can be demonstrated that the MPSE, MLE, PE, and ADE methods can be recommended for estimating the parameters and accelerated factor for the CPALT of Chen distribution when complete data is available.

With the help of the suggested methodology and the results of this investigation, some future studies can be presented, such as:

- Other distributions with different shapes for HRF can be analyzed.
- Additionally, other real data with various phenomena, engineering problems, and clinical treatments can be applied.
- Actually, 6 value sets of the parameters and accelerated factor have been used in the simulation study. Therefore, a number of additional value sets can be used to extend the simulation study and examine their influence on the MSEs and AABs values.

## Data Availability

The data that support the findings of this study are available within the article.
